# Morphology and phylogenetic analysis of five deep-sea golden gorgonians (Cnidaria, Octocorallia, Chrysogorgiidae) in the Western Pacific Ocean, with the description of a new species

**DOI:** 10.3897/zookeys.989.53104

**Published:** 2020-11-09

**Authors:** Yu Xu, Zifeng Zhan, Kuidong Xu

**Affiliations:** 1 Laboratory of Marine Organism Taxonomy and Phylogeny, Institute of Oceanology, Chinese Academy of Sciences, Qingdao 266071, China Institute of Oceanology, Chinese Academy of Sciences Qingdao China; 2 Laboratory for Marine Biology and Biotechnology, Pilot National Laboratory for Marine Science and Technology (Qingdao), Qingdao 266237, China University of Chinese Academy of Sciences Beijing China; 3 Center for Ocean Mega-Science, Chinese Academy of Sciences, Qingdao 266071, China Pilot National Laboratory for Marine Science and Technology Qingdao China; 4 University of Chinese Academy of Sciences, Beijing 100049, China Center for Ocean Mega-Science, Chinese Academy of Sciences Qingdao China

**Keywords:** Anthozoa, *Chrysogorgia dendritica*, *Chrysogorgia carolinensis*, *
Metallogorgia
*, *
Pseudochrysogorgia
*, seamount

## Abstract

Explorations of seamounts in the Western Pacific Ocean and South China Sea resulted in collecting 18 specimens of golden gorgonians. Based on the morphology and the genetic analysis of mtMutS, they are described as one new species, *Chrysogorgia carolinensis***sp. nov.**, and four known species, including *Chrysogorgia dendritica* Xu, Zhan & Xu, 2020, *Metallogorgia melanotrichos* (Wright & Studer, 1889), *Metallogorgia macrospina* Kükenthal, 1919, and *Pseudochrysogorgia bellona* Pante & France, 2010. *Chrysogorgia carolinensis* belongs to the *Chrysogorgia* “group A, Spiculosae” with rods or spindles distributed in the polyp-body wall and tentacles, and differs from all of its congeners except *C. dendritica* by the 1/3L branching sequence and amoeba-shaped sclerites at the basal polyp body. The mtMutS sequence of *C. carolinensis***sp. nov.** has six deletion mutations compared to those of its congeners, supporting the establishment of the new species. Although no genetic variability was observed between the closely related species *C. dendritica* and *C. abludo* Pante & Watling, 2012, the former is different from the latter by the apparently irregular sclerites in the polyp body wall. The two specimens of *Metallogorgia melanotrichos* match well with the original description except for relatively larger polyps, while the *M. macrospina* specimens have slightly smaller polyps than the holotype. The juvenile of *Metallogorgia* has an obvious morphological difference with the adults in the colony shape and branches, but they can be unified by the same polyps and sclerites as well as mitochondrial MutS sequences. Thus, the generic diagnosis of *Metallogorgia* is slightly extended to include the morphology of juveniles. The morphology of *Pseudochrysogorgia bellona* Pante & France, 2010, as a new record for the South China Sea, matches well with that of the original description. In the phylogenetic trees, the *Chrysogorgia* species are separated into two clades, and while *Metallogorgia* and *Pseudochrysogorgia* formed a sister clade.

## Introduction

Chrysogorgiids are found in all major oceans from Iceland to Antarctica, and they are conspicuous members of deep-water benthic assemblages (Pante et al. 2012). The family Chrysogorgiidae Verrill, 1883 currently contains 14 genera, with 12 genera having been analyzed by molecular phylogenetic methods with and six of them forming a monophyletic clade (called the Monophyletic Chrysogorgiidae Clade, or MCC), including *Chrysogorgia* Duchassaing & Michelotti, 1864, *Pseudochrysogorgia* Pante & France, 2010, *Iridogorgia* Verrill, 1883, *Rhodaniridogorgia* Watling, 2007, *Radicipes* Stearns, 1883 and *Metallogorgia* Versluys, 1902 (Watling et al. 2011; Pante et al. 2012).

The genus *Chrysogorgia* Duchassaing & Michelotti, 1864 contains 75 species and is distributed worldwide (Watling et al. 2011; Cairns 2018; Xu et al. 2020). It is characterized by four branching forms of the colony, including a single ascending spiral (clockwise or counterclockwise, bottlebrush-shaped colony), a single fan (planar colony), two or more fans emerging from a short main stem (bi- or multi-flabellate colony) and a bush of branches perched on top of a long straight stem (tree-shaped colony) (Pante and Watling 2012; Cordeiro et al. 2015; Xu et al. 2020). Based on the shapes of rods or scales in the polyp-body wall and tentacles, four groups have been recognized for the separation of *Chrysogorgia* species, including “group A, Spiculosae” with 42 species, “group B, Squamosae aberrantes” with 13 species, “group C, Squamosae typicae” with 19 species and “group D, Spiculosae aberrantes” with only one species (Versluys 1902; Cairns 2001; Cordeiro, Castro and Pérez 2015; Xu et al. 2019).

The genus *Metallogorgia* Versluys, 1902 is distinguished from *Chrysogorgia* by its distinctive monopodial stem with branchlets forming a sympodium pattern in strong branches, sclerites with no distinction in coenenchyme or polyps, and coenenchyme thin with few sclerites (Versluys 1902). The establishment of *Metallogorgia* was well supported by a phylogenetic analysis (Pante et al. 2012). At present, there are four species in this genus: *M. melanotrichos* (Wright & Studer, 1889), *M. macrospina* Kükenthal, 1919, *M. splendens* (Verrill, 1883) and *M. tenuis* Pasternak, 1981 (Verrill 1883; Wright and Studer 1889; Kükenthal 1919; Pasternak 1981; Watling et al. 2011). The genus *Pseudochrysogorgia* Pante & France, 2010 only contains one species *P. bellona* Pante & France, 2010, and has a close relationship with *Metallogorgia* (Pante and France 2010).

During an investigation of the seamount benthic diversity in the Western Pacific Ocean and the South China Sea, we obtained 18 specimens of golden gorgonians. Based on morphological and phylogenetic analyses, they were described as one new species *Chrysogorgia carolinensis* sp. nov., and four known species, including *Chrysogorgia dendritica* Xu, Zhan & Xu, 2020, *Metallogorgia melanotrichos* (Wright & Studer, 1889), *Metallogorgia macrospina* Kükenthal, 1919 and *Pseudochrysogorgia bellona* Pante & France, 2010. Their phylogenetic positions are also discussed.

## Materials and methods

### Specimen collection and morphological examination

One specimen of *Chrysogorgia* Duchassaing & Michelotti, 1864 was collected from a seamount (tentatively named as M2) near the Mariana Trench by the ROV (remotely operated vehicle) *FaXian* (Discovery) in the tropical Western Pacific during the cruises of the R/V *KeXue* (Science) in 2016. One specimen of *Pseudochrysogorgia* Pante & France, 2010 was initially collected from the Ganquan Plateau in the South China Sea in 2018, unfortunately only a few frozen fragments of this specimen and a picture were obtained. Ten specimens of *Chrysogorgia* and six specimens of *Metallogorgia* Versluys, 1902 were obtained from the seamounts (tentatively named as M5–M8) located on the Caroline Ridge during the cruises of the R/V *KeXue* (Science) in 2019. The *Chrysogorgia* and *Metallogorgia* specimens were photographed *in situ* before sampling, and photographed on board and then stored in 75% ethanol after collection. Small branches were detached and stored at -80 °C for molecular study.

The morphological terminology follows Bayer et al. (1983). The general morphology and anatomy were examined by using a stereo dissecting microscope. The sclerites of the polyps and branches were isolated by digestion of the tissues in sodium hypochlorite, and then were washed with deionized water repeatedly. Polyps and sclerites were air-dried and mounted on carbon double adhesive tape and coated for scanning electron microscopy (SEM) observation to investigate their structure. SEM scans were obtained and the optimum magnification was chosen for each kind of sclerite by using TM3030Plus SEM.

The type and voucher specimens have been deposited in the Marine Biological Museum of Chinese Academy of Sciences (**MBMCAS**) at Qingdao, China.

### DNA extraction and sequencing

Total genomic DNA was extracted from the polyps of each specimen using the TIANamp Marine Animal DNA Kit (Tiangen Bio. Co., Beijing, China) following the manufacturer’s instructions. PCR amplification for the mitochondrial genomic region 5’-end of the DNA mismatch repair protein – *mutS* – homolog (mtMutS) was conducted using primers AnthoCorMSH (5’-AGGAGAATTATTCTAAGTATGG-3’; Herrera et al. 2010) and Mut-3458R (5’-TSGAGCAAAAGCCACTCC-3’; Sánchez et al. 2003). PCR reactions were performed as described by Xu et al. (2019). PCR purification and sequencing were performed by TsingKe Biological Technology (TsingKe Biotech, Beijing, China).

### Genetic distance and phylogenetic analyses

The mtMutS gene in octocorals was selected for molecular identification and phylogenetic analyses. All the available mtMutS sequences of *Chrysogorgia*, *Metallogorgia*, *Pseudochrysogorgia* and the out-group species from related chrysogorgiid and plexaurid genera were downloaded from GenBank, and those from duplicate isolates or without associated publications or not identified to species level were omitted from the molecular analyses. To correct possible mistakes, all the selected sequences were visually inspected, and translated to amino acids (AA) to insure all the AA sequences did not include stop codons and suspicious substitutions. The nucleotide and AA sequences were aligned using MAFFT v.7 (Katoh and Standley 2013) with the G-INS-i algorithm. With the guidance of the AA alignment, the nucleotide alignment was refined using BioEdit v7.0.5 (Hall 1999), and only the nucleotide alignment was used in the subsequent analyses. Genetic distances, calculated as uncorrected “p” distances within each species and among species, were estimated using MEGA 6.0 (Tamura et al. 2013).

For the phylogenetic analyses, only one known sequence was randomly selected from the conspecific sequences without genetic divergence (see Table 4). The TPM1uf+G evolutionary model was the best-fitted model for mtMutS, selected by AIC as implemented in jModeltest2 (Darriba et al. 2012). Maximum likelihood (ML) analysis was carried out using PhyML-3.1 (Guindon et al. 2010). For the ML bootstraps, we consider values < 70% as low, 70–94% as moderate and ≥ 95% as high following Hillis and Bull (1993). Node support came from a majority-rule consensus tree of 1000 bootstrap replicates. Bayesian inference (BI) analysis was carried out using MrBayes v3.2.3 (Ronquist and Huelsenbeck 2003) on CIPRES Science Gateway. Posterior probability was estimated using four chains running 10,000,000 generations sampling every 1000 generations. The first 25% of sampled trees were considered burn-in trees. Convergence was assessed by checking the standard deviation of partition frequencies (< 0.01), the potential scale reduction factor (ca. 1.00), and the plots of log likelihood values (no obvious trend was observed over time). For the Bayesian posterior probabilities, we consider values < 0.95 as low and ≥ 0.95 as high following Alfaro et al. (2003). The museum voucher specimen and GenBank accession numbers of the mtMutS sequences were listed next to the species names in the phylogenetic trees (Figure 21).

## Results

### Class Anthozoa Ehrenberg, 1834

**Subclass Octocorallia Haeckel, 1866**

**Order Alcyonacea Lamouroux, 1812**

**Suborder Calcaxonia Grasshoff, 1999**

**Family Chrysogorgiidae Verrill, 1883**

#### Genus Chrysogorgia

Taxon classificationAnimaliaAlcyonaceaChrysogorgiidae

Duchassaing & Michelotti, 1864

F01A7752-D6A1-5019-95FA-A564FB040D0A

##### Diagnosis

**(based on Xu et al. 2020).** Colony branching usually sympodial, occasionally monopodial, arising from a single ascending spiral (clockwise or counterclockwise, bottlebrush-shaped colony), a fan (planar colony), two fans emerging from a short main stem (biflabellate colony), or an unbranched main stem forming a tree-shaped colony. Axis with a metallic shine, dark to golden in color. Branch subdivided dichotomously or pinnately. Most polyps relatively large to the size of the branches they sit on, few in number and well separated from one another. Sclerites in the form of spindles, rods, scales, and rare plates with little ornamentation.

##### Type species.

*Chrysogorgia desbonni* Duchassaing & Michelotti, 1864, by monotypy.

##### Distribution.

Worldwide in a depth range of 10–4492 m (Watling et al. 2011).

#### Chrysogorgia dendritica

Taxon classificationAnimaliaAlcyonaceaChrysogorgiidae

Xu, Zhan & Xu, 2020

105222F6-271F-5F64-8AD5-C6EAA58B8402

**Figure 1.**
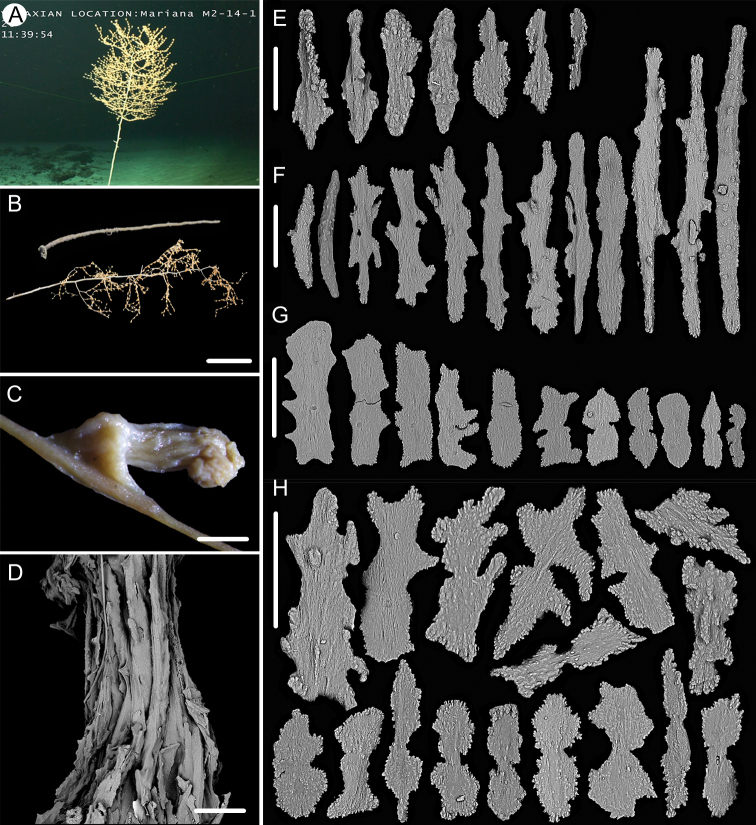
External morphology, polyps and sclerites of *Chrysogorgia dendritica* MBM286353 **A, B** the tree-shaped colony *in situ* and after collection (likely an adult), with a broken stem and a branching part **C** a single polyp under a light microscope **D** long polyp neck under SEM**E** sclerites in tentacles **F** sclerites of the polyp neck **G** sclerites in coenenchyme **H** sclerites at the basal polyp body. Scale bars: 10 cm (**B**); 1 mm (**C**); 200 μm (**D**); 50 μm (**E**), 100 μm (**F, G, H**).

**Figure 2.**
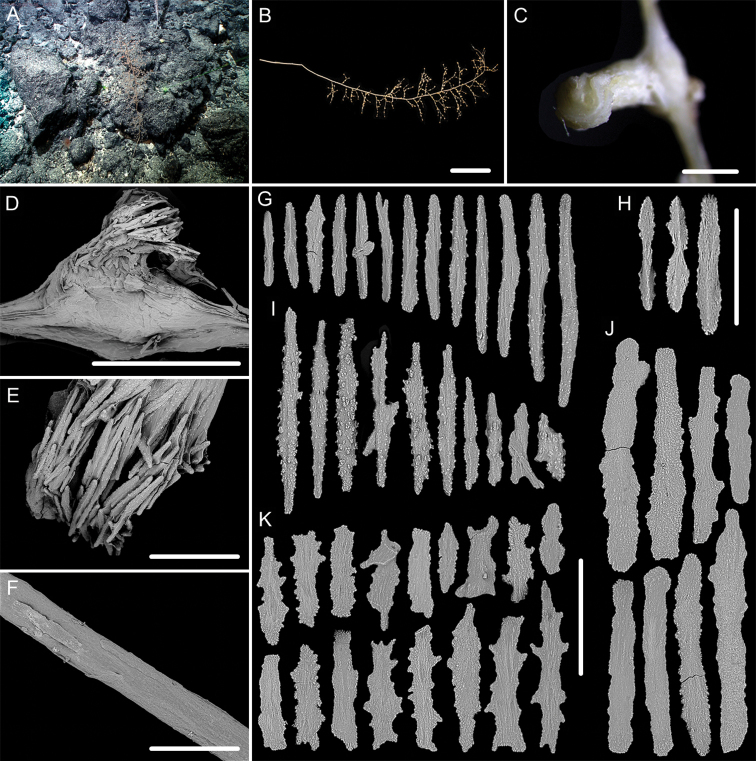
External morphology, polyps and sclerites of *Chrysogorgia dendritica* MBM286442 **A, B** the bottlebrush-like colony *in situ* and after collection (likely a juvenile) **C** a single polyp under a light microscope **D** a single polyp under SEM**E** tentacular part under SEM**F** coenenchyme under SEM**G** sclerites in tentacles **H** sclerites extending to pinnules **I** sclerites of the polyp neck **J** sclerites in coenenchyme **K** sclerites at the basal polyp body. Scale bars: 10 cm (**B**); 1 mm (**C, D**); 300 μm (**E, F**); 200 μm (**G, I–K** at the same scale), 100 μm (**H**).

**Figure 3.**
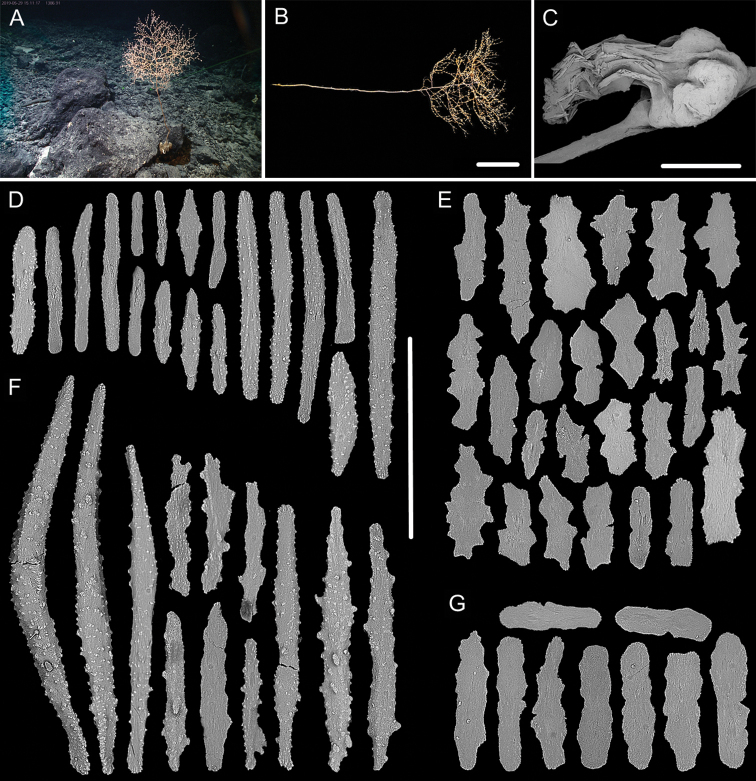
External morphology, polyps and sclerites of *Chrysogorgia dendritica* MBM286443 **A, B** the tree-shaped colony *in situ* and after collection (likely an adult) **C** a single polyp under SEM**D** sclerites in tentacles **E** sclerites at the basal polyp body **F** sclerites of the polyp neck **G** sclerites in coenenchyme. Scale bars: 10 cm (**B**); 1 mm (**C**); 300 μm (**D–G** at the same scale).

**Figure 4.**
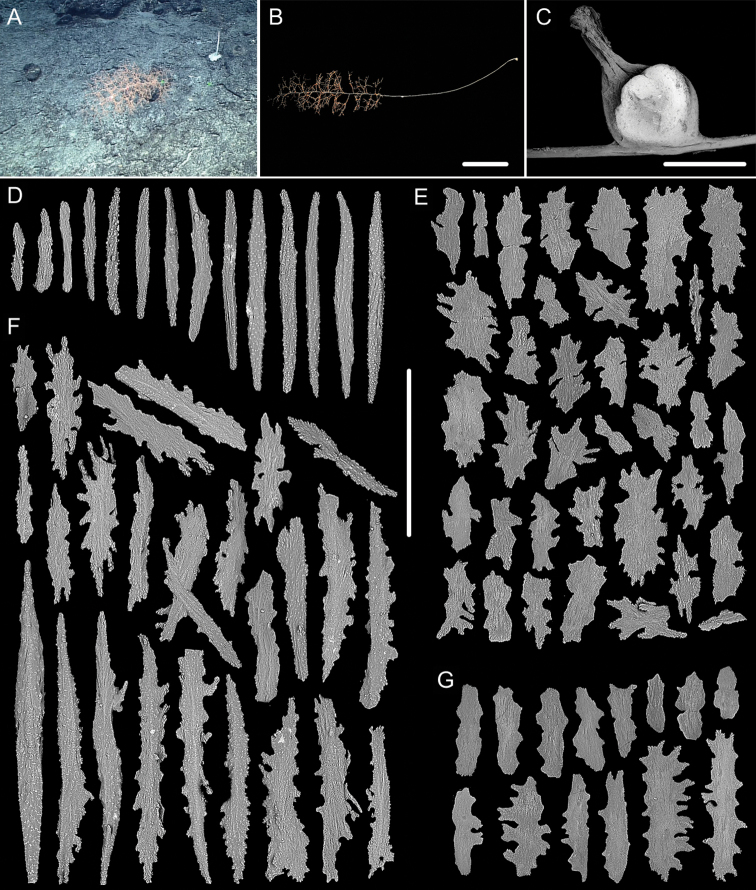
External morphology, polyps and sclerites of *Chrysogorgia dendritica* MBM286444 **A, B** the colony *in situ* and after collection (likely an intermediate state) **C** a single polyp under SEM**D** sclerites in tentacles **E** sclerites of the polyp neck **F** sclerites at the basal polyp body **G** sclerites in coenenchyme. Scale bars: 20 cm (**B**); 2 mm (**C**); 300 μm (**D–G** at the same scale).

Figures 1 , 2 , 3 , 4 ; Table 1

Chrysogorgia dendritica Xu, Zhan & Xu, 2020: 6–8, figs 2, 3.

##### Type locality.

Kocebu Guyot in the Magellan Seamount chain, 1821 m depth.

**Voucher specimens.** MBM286353, station FX-Dive 71 (11°20.83'N, 139°15.87'E), a seamount (tentatively named as M2) near the Mariana Trench, depth 1375 m, 28 March 2016. MBM286442, station FX-Dive 211 (10°02.97'N, 140°10.48'E), a seamount (tentatively named as M5) located on the Caroline Ridge, depth 1475 m, 29 May 2019. MBM286443, station FX-Dive 211 (10°03.27'N, 140°10.70'E), a seamount (M5) located on the Caroline Ridge, depth 1387 m, 29 May 2019. MBM286444, station FX-Dive 227 (10°37.92'N, 140°05.62'E), a seamount (tentatively named as M8) located on the Caroline Ridge, depth 1702 m, 15 June 2019. GenBank accession number: MT269888.

##### Extended diagnosis.

*Chrysogorgia* “group A, Spiculosae” with 1/3L Branching sequence and a monopodial or a little zigzagging stem. Juvenile with a bottlebrush-like colony, while adult usually having a tree-shaped colony. Branches nearly perpendicular to stem, subdivided dichotomously. Polyps with a long neck and an expanded base. Rods and rare scales in tentacles, longitudinally arranged. Rods/spindles and elongate scales in polyp neck longitudinally arranged, coarse with many warts on surface. Scales and rare plates at the basal polyp body irregularly and alternately arranged, irregular and often amoeba-shaped. Scales in coenenchyme sparse and elongate, usually lobed with irregular edges.

##### Description.

For morphological measurements, see Table 1.

##### Distribution.

Kocebu Guyot, 1821 m (Xu et al. 2020); a seamount adjacent to the Mariana Trench and seamounts on the Caroline Ridge, 1375–1702 m depth.

##### Remarks.

The four specimens match the holotype of *Chrysogorgia dendritica* Xu, Zhan & Xu, 2020 in having a monopodial stem and the same sclerite form, for example, rods and rare scales in tentacles, rods/spindles and elongate scales in polyp neck, irregular scales at the basal polyp body, and elongate scales in coenenchyme. Moreover, their mtMutS gene sequences are identical (see the genetic analysis below). Thus, we identified the four specimens as *C. dendritica*. The sclerites in the four voucher specimens and the holotype showed some differences: (1) rod-like scales with an obvious medial contraction often present in MBM286353 and MBM286442, while rare in the holotype, MBM286443 and MBM286444 (Figures 1A, 2H vs. Figures 3D, 4D); (2) large cured spindles are rarely present in the polyp neck of the holotype, MBM286443 and MBM286444, while absent in the other two specimens (Figures 3F, 4F vs. Figures 1F, 2I); (3) scales at the basal polyp body of the holotype and MBM286444 are more irregular and amoeba-shaped than the other specimens (Figure 4E vs. Figures 1H, 2K, 3E); and (4) scales in coenenchyme are more elongate in MBM286442 than the other specimens (Figure 2J vs. Figures 1G, 3G, 4G). However, these differences are minor and not constant, and we thus treate as the conspecific variation.

The four specimens of *C. dendritica* showed a series of growth stages, from bottlebrush-like colony (juvenile) to tree-shaped colony (adult). Considering the diameter size of the stem base and scars on the stem, the specimen MBM286442 is likely a juvenile with a narrow stem and without scars, while the other specimens have wider stems and some old scars of the past branches (Table 1). The juvenile has a bottlebrush-like colony (Figure 2B), while the adult has a long monopodial stem with branches occurring on the top, forming a tree-shaped colony (Figures 1B, 3B). Compared to the changing colony shapes, the sclerite forms showed small variation in the growth stages and can be used as a main character to identify the species.

#### Chrysogorgia carolinensis sp. nov.

Taxon classificationAnimaliaAlcyonaceaChrysogorgiidae

5F35D75E-99BE-54BE-8853-99BA426EC474

http://zoobank.org/A37B30BA-6396-4679-9D39-1FFB51FCCD4D

**Figure 5.**
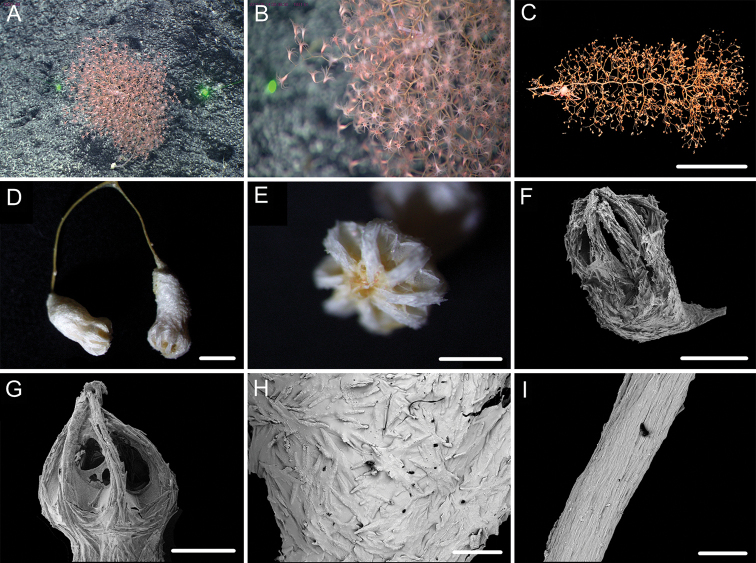
External morphology and polyps of *Chrysogorgia carolinensis* sp. nov. **A** the holotype *in situ***B** close-up branches *in situ***C** the holotype after collection **D** two polyps under a light microscope **E** tentacles under a light microscope **F** a single polyp under SEM**G** upper part of a polyp under SEM**H** basal polyp body under SEM**I** coenenchyme under SEM. Scale bars: 10 cm (**B**); 2 mm (**D**); 1 mm (**E–G**); 300 μm (**H**); 100 μm (**I**).

**Figure 6.**
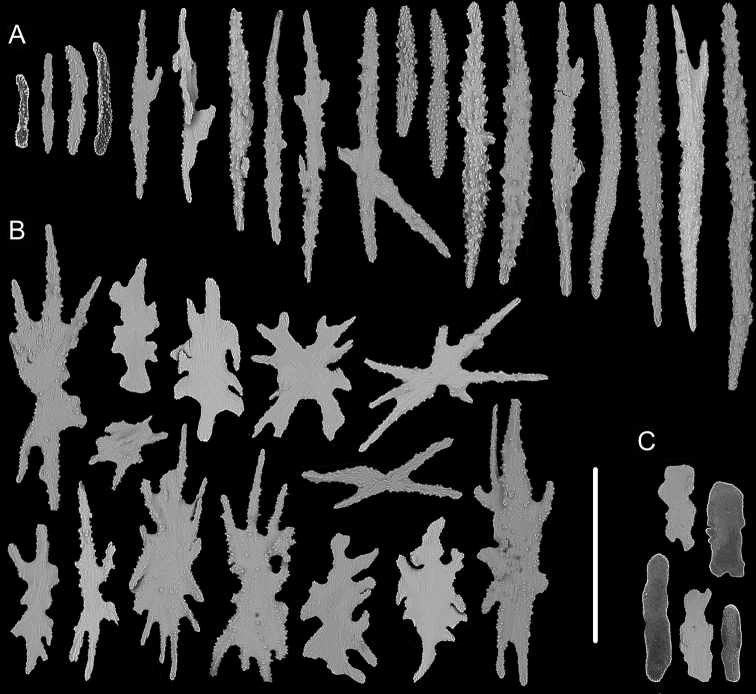
Sclerites of *Chrysogorgia carolinensis* sp. nov. **A** sclerites in the back and at the base of tentacles **B** sclerites at the basal polyp body **C** sclerites in coenenchyme. Scale bar: 300 μm (all images at the same scale).

Figures 5 , 6 ; Table 1

##### Type material.

***Holotype*.** MBM286494, station FX-Dive 226 (10°38.18'N, 140°04.08'E), a seamount (tentatively named as M8) located on the Caroline Ridge, depth 1832 m, 14 June 2019. GenBank accession number: MT269889.

***Paratypes*.** MBM286493, station FX-Dive 224 (10°37.63'N, 140°05.45'E), depth 1509 m, 12 June 2019. MBM286495, station FX-Dive 227 (10°37.92'N, 140°05.62'E), depth 1709 m. MBM286496, station FX-Dive 227 (10°37.92'N, 140°05.62'E), depth 1706 m, 15 June 2019. MBM286497, station FX-Dive 227 (10°37.90'N, 140°05.62'E), depth 1695 m, 15 June 2019. MBM286498, station FX-Dive 227 (10°37.68'N, 140°05.48'E), depth 1537m, 15 June 2019. MBM286499, station FX-Dive 227 (10°37.60'N, 140°05.43'E), depth 1506 m, 15 June 2019. They were all collected from a seamount (tentatively named as M8) located on the Caroline Ridge.

##### Diagnosis.

*Chrysogorgia* “group A, Spiculosae” with 1/3L branching sequence. Branches subdivided dichotomously, up to sixth order. Polyps only present in the end of terminal branchlets. Polyps large with pitcher shape, up to 8 mm long. Rods and spindles slender and coarse with many warts on surface in the back and base of tentacles. Scales amoeba-shaped, branched toward to any directions, irregularly and alternately arranged at basal polyp body. Scales rare, transversally arranged in coenenchyme.

##### Description.

Specimen of holotype ca. 31 cm long and 14 cm wide excluding the holdfast (Figure 5C). Colony bottlebrush-shaped, with branching sequence 1/3L. Stem golden with metallic luster, ca. 1 mm in diameter at base. Branches subdivided dichotomously, up to sixth order, with distance between adjacent branches 6–12 mm long and orthostiche interval 19–35 mm long in measurements from all specimens. Branches up to 6 cm with the first branch internodes 7–15 mm long and terminal branchlets up to 2 cm long. Polyps only present in the end of the terminal branchlets. Polyps large with pitcher shape, some of them contracted and narrow at the base of tentacles, average 5 mm long with the terminal one up to 8 mm long, and 1–3 mm wide (Figure 5B, D, F). No polyps in axis internodes.

Rods and spindles slender and coarse with many warts on surface, some of them branched, rarely with one end a little flat, longitudinally arranged in the back of tentacles and usually forming eight distinct columns, and transversally or longitudinally arranged in the base of tentacles, measuring 107–814 × 10–78 μm (length × width, the same below, Figures 5E, G, 6A). Rare sclerites extend into pinnules, but mostly pinnules free of sclerites. Scales amoeba-shaped, branched in any directions, irregularly and alternately arranged at the basal polyp body, and measuring 161–483 × 14–170 μm (Figures 5H, 6B). Scales rare and elongate, some of them lobed with irregular edges, transversally arranged in coenenchyme, and measuring 139–221 × 29–67 μm (Figures 5I, 6C).

##### Type locality.

A seamount (tentatively named as M8) located on the Caroline Ridge with a depth range of 1506–1832 m.

##### Etymology.

Named after the type locality, the Caroline Ridge, where the species was discovered.

##### Distribution and habitat.

Found only from a seamount located on the Caroline Ridge. Colony attached to a rocky substrate (Figure 5A).

##### Remarks.

*Chrysogorgia carolinensis* sp. nov. belongs to the “group A, Spiculosae” with an unusual branching sequence of 1/3L and bottlebrush-shaped colony, which is similar to *C. midas* Cairns, 2018 and *C. abludo* Pante & Watling, 2012. However, the new species differs distinctly from these species by the presence of amoeba-shaped scales, which branch toward to any directions at the basal polyp body (vs. absence in both species). *Chrysogorgia carolinensis* sp. nov. is also similar to *C. dendritica* by 1/3L branching sequence and the conspicuously amoeba-shaped sclerites at the basal polyp body. However, the new species is easily separated by the bottlebrush-shaped colony (vs. tree-shaped), absence of polyps on internodes (vs. presence) and larger polyps up to 8 mm long (vs. no more than 5 mm) (Table 1).

**Table 1.** Morphological comparisons between *Chrysogorgia carolinensis* sp. nov., *Chrysogorgia dendritica* Xu, Zhan & Xu, 2020 and *Chrysogorgia abludo* Pante & Watling, 2012, including detailed morphological measuring data of five specimens of *C. dendritica*.

Characters/species	*Chrysogorgia dendritica*					*Chrysogorgia carolinensis* sp. nov.	*Chrysogorgia abludo*	
Specimen	holotype	MBM286353	MBM286442	MBM286443	MBM286444	holotype	holotype	paratype
Group type	A					A	A	
Branching sequence	1/3L					1/3L	1/3, 1/4L, irregular	
Axis	monopodial, or a little zigzagging					sympodial	monopodial	
Colony shape	tree-shaped	tree-shaped	bottlebrush-like	tree-shaped	a little tree-shaped	bottlebrush-shaped	bottlebrush-shaped	tree-shaped
Colony height (cm)	57	85.5	55	50	110	31	16	50
Basal stem width (mm)	2	7	1.2	3	3	1	No data	2.2
Interbranch distance (mm)	16–22	18	5–22	11–15	12–24	6–12	4.3–6.8	7.5–15.0
Orthostiche interval (mm)	50–55	50–55	37–47	36–47	44–61	19–35	No data	No data
First branch internode (mm)	15–20	14–22	11–20	9–23	20–30	7–15	6.1–11.0	16
Polyps on internodes	1–5	2–3	1–3	1–4	1–4	0	1–2	No data
Polyps on terminal branchlets	1–6	1–4	1–4	1–8	1–8	1	1–3	1–6
Polyps height (mm)	3	3–5	1.5–2.0	2–3	3–4	3–8, average 5	0.8–2.2	0.8–2.2
Sclerites in coenenchyme (μm)	flat elongate scales often with lobed edges	82–200 × 10–96	139–420 × 16–112	92–266 × 15–57	53–379 × 10–34	elongate scales occasionally with lobed edges	small rugged scales with less lobed edges	
Sclerites in body wall (μm)	scales, rods and spindles	68–190 × 7–58 at basal body; 171–516 × 20–55 in neck	100–306 × 20–72 at basal body; 121–353 × 12–56 in neck	80–229 × 13–82 at basal body; 207–614 × 14–76 in neck	39–236 × 8–128 at basal body; 137–590 × 12–98 in neck	scales, rods and spindles	scales and rods	
Sclerites in tentacles (μm)	scales and rods	74–135 × 4–32	105–365 × 6–45	75–429 × 10–43	93–275 × 24–93	rods and spindles	rods	
Distribution	Kocebu Guyot	an unnamed seamount adjacent to the Mariana Trench	unnamed seamounts on the Caroline Ridge			an unnamed seamount on the Caroline Ridge	North Atlantic	
References	Xu et al. 2020	Present study				Present study	Pante and Watling 2012	

#### Genus Metallogorgia

Taxon classificationAnimaliaAlcyonaceaChrysogorgiidae

Versluys, 1902

30173370-1C8E-531B-9F47-FC99F5C9FB0E

##### Diagnosis

**(based on Versluys 1902; Nutting 1908; Kükenthal 1919).** Main stem monopodial with a few branches occurring on the side. Axis round and solid with a smooth surface and strong metallic luster. Branches irregular, subdivided dichotomously with branchlets forming a sympodium. The coenenchyme usually thin with a few sclerites or not well differentiated this layer.

##### Type species.

*Metallogorgia melanotrichos* (Wright & Studer, 1889)

##### Distribution.

Atlantic Ocean, Pacific Ocean and Indian Ocean, in a depth range of 567–2311 m (Watling et al. 2011, and the present study).

#### Metallogorgia melanotrichos

Taxon classificationAnimaliaAlcyonaceaChrysogorgiidae

(Wright & Studer, 1889)

719D0488-5A1E-57D3-A25F-DBDB30A9D48C

**Figure 7.**
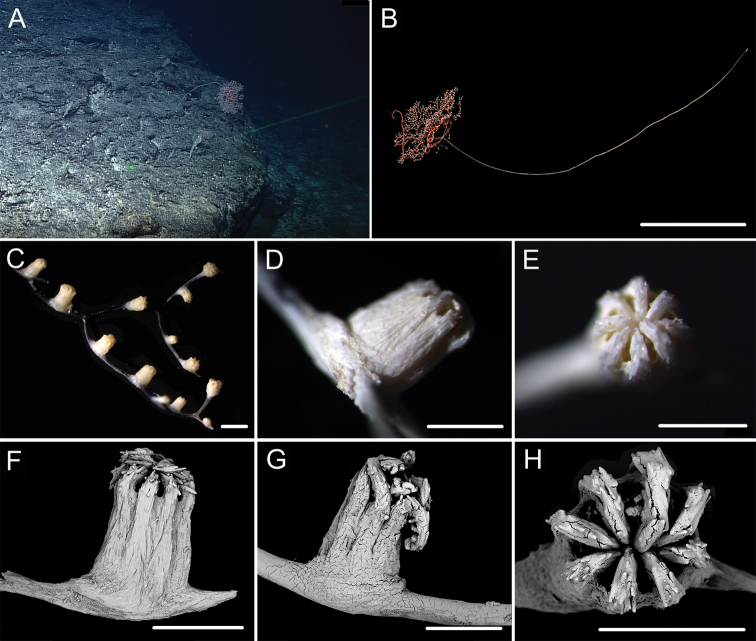
External morphology and polyps of *Metallogorgia melanotrichos* MBM286485 **A** the colony *in situ*. Laser dots spaced at 33 cm used for scale **B** the colony after collection **C** a branch under a light microscope **D, E** a single polyp under a light microscope **F, G** a single polyp under SEM**H** head of one polyp under SEM. Scale bars: 20 cm (**B**), 2 mm (**C**), and 1 mm (**E–H**).

**Figure 8.**
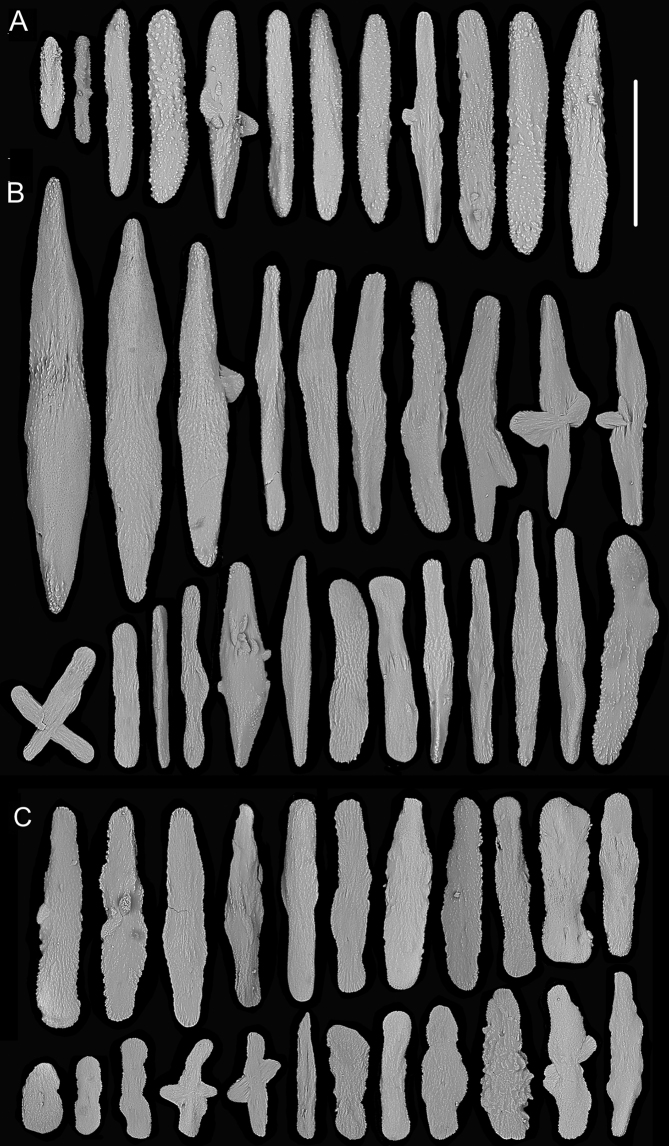
Sclerites of *Metallogorgia melanotrichos* MBM286485 **A** rods in tentacles **B** rods and scales in the polyp-body wall **C** scales in coenenchyme. Scale bar: 200 μm (all images at the same scale).

**Figure 9.**
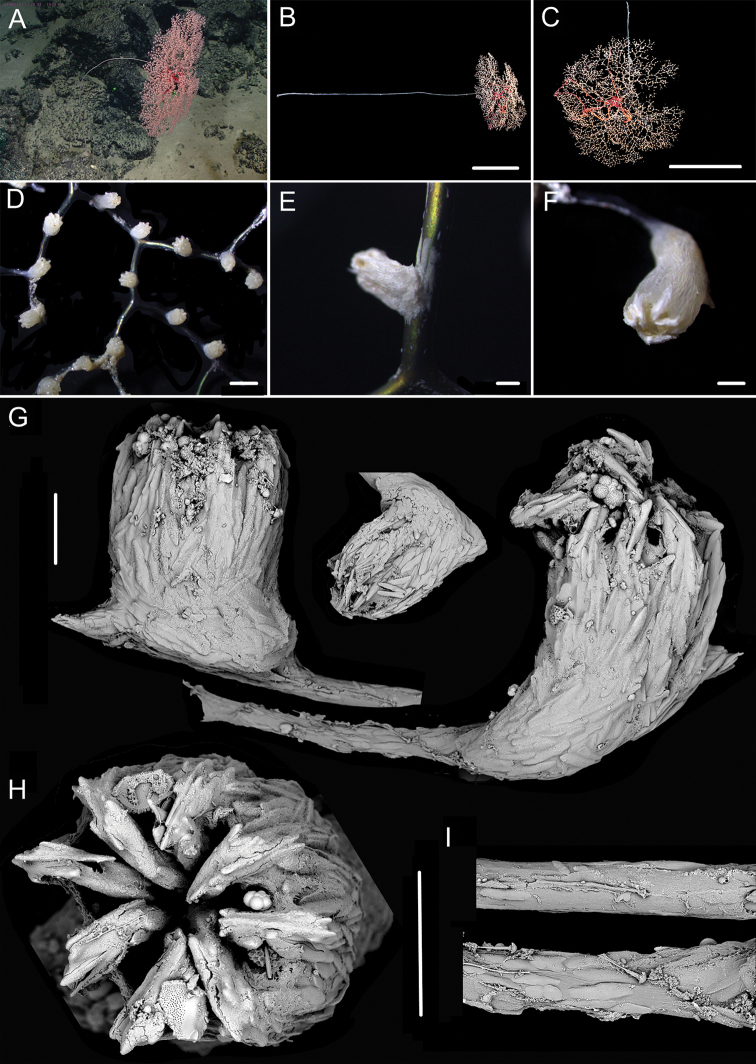
External morphology and polyps of *Metallogorgia melanotrichos* MBM286486 **A** the colony *in situ*. Laser dots spaced at 33 cm used for scale **B, C** the colony after collection **D** a branch under a light microscope **E, F** a single polyp under a light microscope **G** three polyps under SEM**H** head of one polyp under SEM**I** coenenchyme under SEM. Scale bars: 10 cm (**B, C**), 2 mm (**D**) and 500 μm (**E–I; H, I** at the same scale).

**Figure 10.**
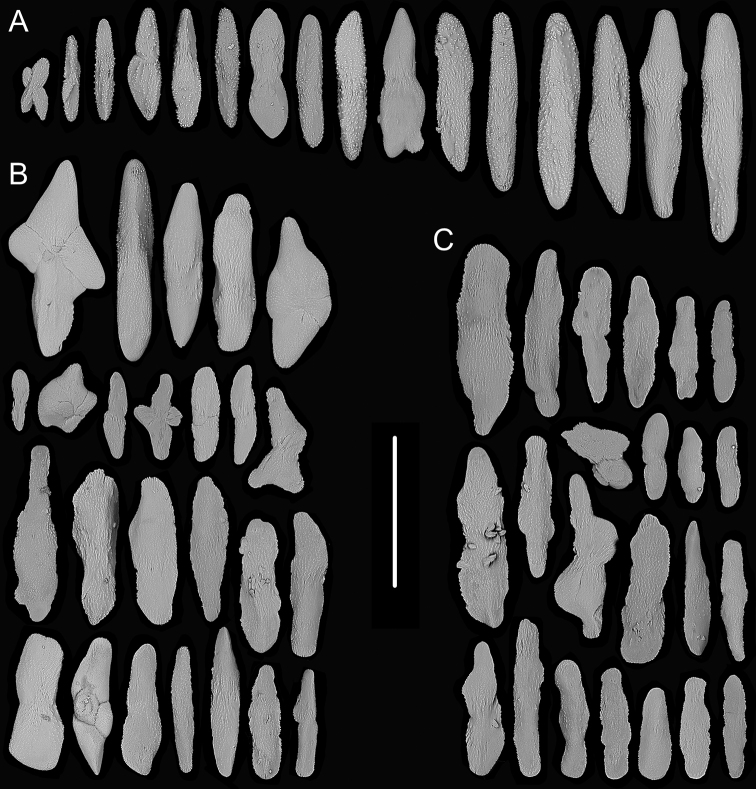
Sclerites of *Metallogorgia melanotrichos* MBM286486 **A** rods in tentacles **B** rods and scales in the polyp-body wall **C** scales in coenenchyme. Scale bar: 300 μm (all images at the same scale).

Figures 7 , 8 , 9 , 10 ; Tables 2 , 3

Dasygorgia melanotrichos Wright & Studer, 1889: 15, pl. IV, fig. 3, pl. V, fig. 5.
Metallogorgia melanotrichos : Versluys, 1902: 87.
Metallogorgia melanotrichos : Nutting, 1908: 593–594, pl. LI, fig. 5.
Metallogorgia melanotrichos : Kükenthal, 1919: 503.
Metallogorgia melanotrichos : Pasternak, 1981: 51.

##### Type locality.

Ascension Island in the South Atlantic Ocean, 778 m depth (Wright and Studer 1889).

##### Voucher specimens.

MBM286485, station FX-Dive 222 (10°04.73'N, 140°09.45'E), depth 1839 m, 10 June 2019; MBM286486, station FX-Dive 227 (10°37.92'N, 140°05.62'E), depth 1706 m, 15 June 2019. They were collected from two seamounts (tentatively named as M5 and M8, respectively) located on the Caroline Ridge in the West Pacific Ocean.

##### Diagnosis

**(extended on the basis of Versluys 1902; Kükenthal 1919; Mosher and Watling 2009).** In adults, main stem monopodial with a few large branches occurring on the distal end. Branching angle between these large branches usually obtuse. In each large branch, strong branchlets subdivided dichotomously and forming a sympodium pattern. In juveniles, main stem monopodial and gracile with branches producing on the lateral of the trunk randomly and subdivided dichotomously in multiple planes. Polyps conical or cylindrical, absent on the stem of adults but present in juveniles. Sclerites elongated and often crossed, arranged densely in polyps and relatively sparsely in coenenchyme. Rods longitudinally arranged in tentacles, covered with sparse fine warts. Rods longitudinally arranged on upper part of polyp body, and scales partially crosswise or transversely arranged on bottom, with nearly smooth surface. Scales transversely arranged in coenenchyme, usually with rounded ends and occasionally irregular edges. Nematozooids absent.

##### Description.

For morphological measurements, see Table 2.

**Table 2.** The morphological measuring data of the specimens of *Metallogorgia*. “–” means nonexistent or meaningless data.

Characters/ Specimens	MBM286485	MBM286486	MBM286484	MBM286487	MBM286488	MBM286489
Species	*M. melanotrichos*	*M. melanotrichos*	*M. macrospina*	*M. macrospina*	*M. macrospina*	*M. macrospina*
Colony height (cm)	76	35	56	51	69	48
Basal stem width (mm)	2.7	1.5	1.0	2.5	3.0	2.5
Branching part height (cm)	9	8	–	12	11	7
Branching part width (cm)	18	18	6	26	20	14
Branch maximal length (cm)	10	12	4	16	13	12
Branches	2	2	22	14	7	6
Interbranch distance (mm)	–	–	9–25	6–13	13–18	5–18
Internode length (mm)	4–12	3–11	3–7	3–8	3–10	3–11
First internode length (mm)	13–24	12–18	3–8	5–8	6–8	6–11
Polyp height (mm)	1.0–2.5	1.0–4.0, most 2.0	1.0–2.5, most 1.0	average 1.0	1.0–1.5	1.0–1.5
Polyps in internode	1, rarely 2	1, rarely 2	1, rarely 2	1, rarely 2	1, rarely 2	1, rarely 2
Polyps in terminal branchlets	1–3	1–4	1–4	1–4	1–5	1–2
Inter-polyp distance (mm)	1–6	1–4	2–5	1–3	0–5	0–4
Sclerites measured in tentacles (μm)	129–433 × 17–55	125–467 × 21–125	96–450 × 10–85	44–542 × 6–117	120–492 × 12–98	115–460 × 14–78
Sclerites measured in body wall (μm)	172–571 × 14–93	128–429 × 25–125	96–450 × 10–85	44–542 × 6–117	137–473 × 17–93	57–417 × 11–66
Sclerites measured in coenenchyme (μm)	96–378 × 18–66	148–379 × 31–113	112–456 × 10–62	175–394 × 17–75	78–328 × 11–51	70–372 × 14–50
Figures	7, 8	9, 10	11, 12	13, 14	15, 16	17, 18

##### Distribution.

Central Indo-Pacific Ocean (Wright and Studer 1889; Versluys 1902), Western and Central Pacific (Nutting 1908; Pasternak 1981; present study), Atlantic Ocean, 183–2265 m depth (Kükenthal 1919; Watling et al. 2011; Pante et al. 2012).

##### Remarks.

*Metallogorgia melanotrichos* (Wright & Studer, 1889) is characterized by its completely monopodial stem, with the branches in adults occurring on the distal end, and scales in both body wall and coenenchyme (Table 3). Furthermore, *M. melanotrichos* has a much more extensive distribution than its congeners, while other species have a relatively limited distribution. Our specimens match well with the original description in the sclerites, but possess a relatively larger polyp (most 2 mm, up to 4 mm vs. 1.75 mm), and longer scales in coenenchyme (up to 379 μm vs. 225 μm) (Kükenthal 1919, Table 2). The branches of the specimen MBM286486 on one side is irregular and tend to form a fake spiral with branches subdivided dichotomously in multiple planes, while it is regular and forms a planar layer that nearly perpendicular to the trunk in the specimen MBM286485.

**Table 3.** The comparisons of the species (adults) in the genus *Metallogorgia* Versluys, 1902 and *Pseudochrysogorgia bellona* Pante & France, 2010.

Characters/ Species	*M. macrospina*	*M. melanotrichos*	M. splendens*	*M. tenuis*	*P. bellona*
Branching part	sympodial	monopodial	unknown	sympodial*	monopodial
Branchlets forming a sympodium	yes	yes	unknown	No*	No
Interbranch distance (mm)	5–18	–	unknown	10–18*	9.9–17.2
Polyp height (mm)	1–1.5	1.5–2.0	1.5	1.5–2.0	1.0–3.3
Sclerites in tentacles	rods	rods	plates	spindles	rods
Sclerites in body wall	rods	rods and scales	rods	rods and spindles	rods and scales
Sclerites with its shapes in coenenchyme	rods and scales elongated with slightly serrated edges and coarse surface	scales elongated with relatively smooth edges and surface	rods small, flattened, often with serrated edges and smooth surface	scales small, elongated, often with irregular shape and serrated edges.	scales and plates with many ornamentation
Dendritic holdfast	no	no	unknown	no	yes
Reference	Kükenthal 1919, present study	Wright and Studer 1889, Versluys 1902, Nutting 1908, Kükenthal 1919, Pasternak 1981, present study	Verrill 1883, Versluys 1902, Kükenthal 1919, Deichmann 1936	Pasternak 1981	Pante and France 2010, present study

*, putative species or questionable regarded by Watling et al. 2011.

#### Metallogorgia macrospina

Taxon classificationAnimaliaAlcyonaceaChrysogorgiidae

Kükenthal, 1919

FFA4B27A-101A-5FA7-9F6C-E9821D8E2C5A

Figures 11 , 12 , 13 , 14 , 15 , 16 , 17 , 18 ; Tables 2 , 3

Metallogorgia macrospina Kükenthal, 1919: 504–505, figs 227–229, Taf.XXX, Fig. 6.

##### Type locality.

0°58.2'S, 90°43.2'E, West Sumatra, 1280 m depth (Kükenthal 1919).

##### Voucher specimens.

MBM286484, station FX-Dive 210 (10°04.68'N, 140°12.07'E), depth 911 m, 28 May 2019. MBM286487, station FX-Dive 215 (10°04.97'N, 140°10.75'E), depth 986 m, 2 June 2019. MBM286488, station FX-Dive 215 (10°04.82'N, 140°10.90'E), depth 902 m, 2 June 2019. MBM286489, station FX-Dive 223 (10°04.63'N, 140°15.12'E), depth 1072 m, 11 June 2019. They were collected from three seamounts (tentatively named as M5, M7 and M8) located on the Caroline Ridge.

##### Diagnosis

**(extended on the basis of Kükenthal 1919).** In adults, main stem monopodial with branches forming a similar spiral on the top. Strong branches subdivided dichotomously, with branchlets forming a sympodium pattern in each plane. In juveniles, main stem monopodial and thin/gracile with branches occurring on the lateral of the trunk randomly and subdivided dichotomously in multiple planes. Polyp cylindrical, some of them with a slightly expanded base, absent on stem of adults but present in juveniles. Sclerites relatively coarse with many small warts on surface, cross-shaped occasionally. Rods relatively regular, longitudinally arranged in tentacles and the upper part of the polyp body, and partially crosswise or transversely arranged on the body bottom. Scales and rods elongated, usually coarse with serrated edges, transversely arranged in coenenchyme. Nematozooids not present.

##### Description.

In juvenile (specimen MBM286484), colony slightly bottlebrush-shaped, with branches occurring on the lateral side of the stem randomly. Main stem monopodial and gracile. Branches subdivided dichotomously in multiple planes. Polyps cylindrical, occasionally present on the top of the stem. The branch coenenchyme well differentiated with a layer of sclerites. In the adults (specimen MBM286487–286489), colony like a tree shape with branches forming a similar spiral on the top of the stem. Main stem monopodial and strong. Branches subdivided dichotomously with branchlets forming a sympodium pattern in each plane. Polyps cylindrical, some of them with a slightly expanded base, absent from the stem. Branch coenenchyme usually not well differentiated.

Sclerites with same forms and arrangement in juveniles and adults, both relatively coarse with many small warts on surface, cross-shaped occasionally. Rods relatively regular, longitudinally arranged in tentacles and the upper part of the polyp body, and partially crosswise or transversely arranged on the body bottom. Scales and rods elongated, usually coarse with serrated edges, transversely arranged in coenenchyme. For detailed morphological measurements, see Table 2.

##### Distribution.

West Sumatra (Kükenthal 1919); the unnamed seamounts on the Caroline Ridge in the Western Pacific (present study); Southwest Pacific (Pante et al. 2012), 720–1280 m depth.

##### Remarks.

According to Kükenthal (1919), the sclerites in the *M. macrospina* polyps contain rods and spindles, and those in coenenchyme are slender rods, some of them flat or irregular. The sclerites in our specimens match well with the original description as well as pictures. Therefore, we identify our specimen as *M. macrospina*.

*Metallogorgia macrospina* is similar to *M. melanotrichos* by the branchlets forming a sympodium pattern in the large branches. In the original description, Kükenthal (1919) pointed out that *M. macrospina* differed from *M. melanotrichos* by its more densely arranged branches, larger polyps, longer sclerites in coenenchyme and different color. However, the polyps of *M. macrospina* in our specimens are generally smaller than those of *M. melanotrichos* (Table 2), and there are no conspicuous differences in color (almost brown to black) among these colonies. Therefore, based on the morphological features of our specimens, *M. macrospina* can be separated from *M. melanotrichos* by its sympodial branching part forming a spiral on the colony top (vs. monopodial), only rods in the polyp-body wall (vs. rods and scales), and rods and scales in coenenchyme (vs. only scales).

The juvenile of *Metallogorgia macrospina* (specimen MBM286484) has significant differences in the branching pattern from the adult specimens MBM286487, MBM286488 and MBM286489 in morphology. It differs also by having a slightly bottlebrush-shaped colony (vs. similarly tree-shaped colony), gracile and flexible stem (vs. hard and strong), monopodial branching part (vs. sympodial), branchlets in multiple planes (vs. forming a sympodium in one plane), and well differentiated coenenchyme with more sclerites (vs. not well differentiated and with relatively sparse sclerites). However, the same sclerites in polyps and coenenchyme, and particularly the mtMutS gene data analyzed below indicate these specimens belong to the same species.

**Figure 11.**
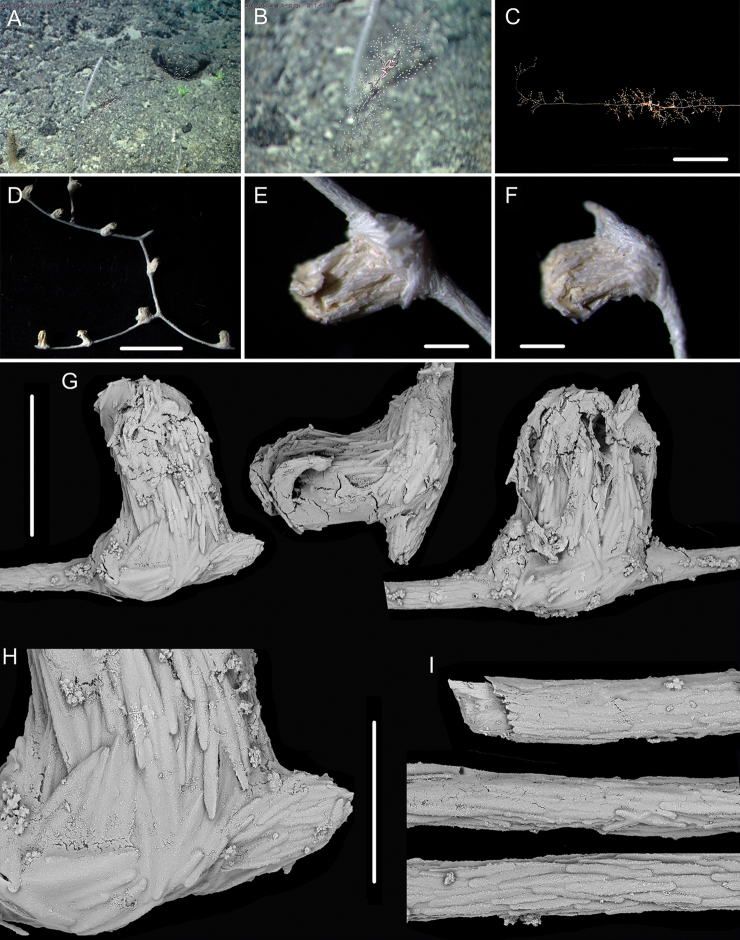
External morphology and polyps of juvenile *Metallogorgia macrospina* MBM286484 **A** the colony *in situ*. Laser dots spaced at 33 cm used for scale **B** close-up of branches *in situ***C** the colony after collection **D** a branch under a light microscope **E, F** a single polyp under a light microscope **G** three polyps under SEM**H** polyp-body wall under SEM**I** coenenchyme under SEM. Scale bars: 10 cm (**C**), 5 mm (**D**), 1 mm (**G**), and 500 μm (**E, F, H, I; H, I** at the same scale).

**Figure 12.**
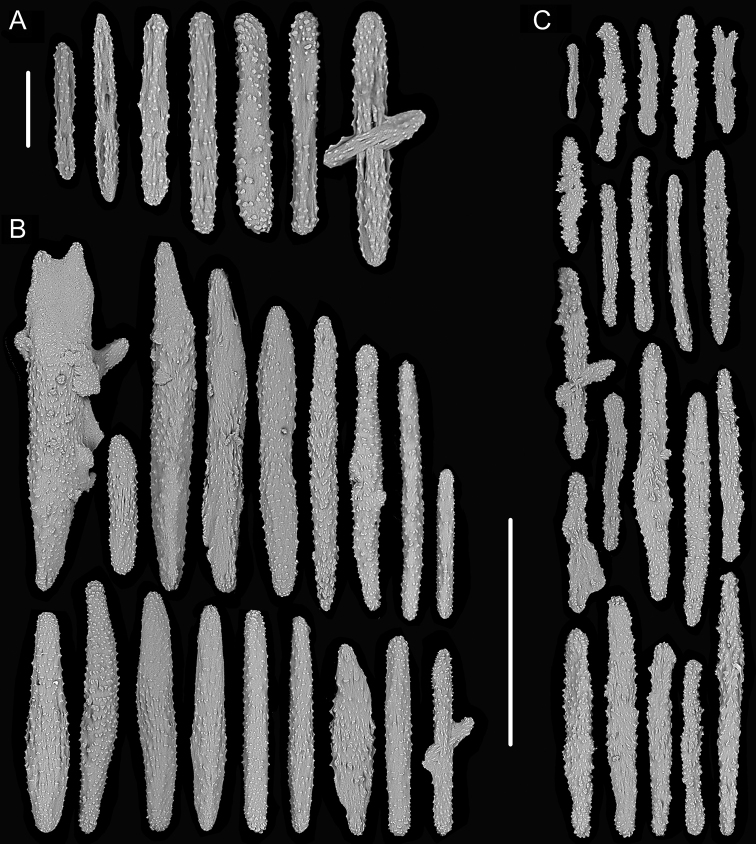
Sclerites of juvenile *Metallogorgia macrospina* MBM286484 **A** some small rods in polyps **B** larger rods in polyps **C** rods and elongated scales in coenenchyme. Scale bars: 50 μm (**A**), 300 μm (**B, C** at the same scale).

**Figure 13.**
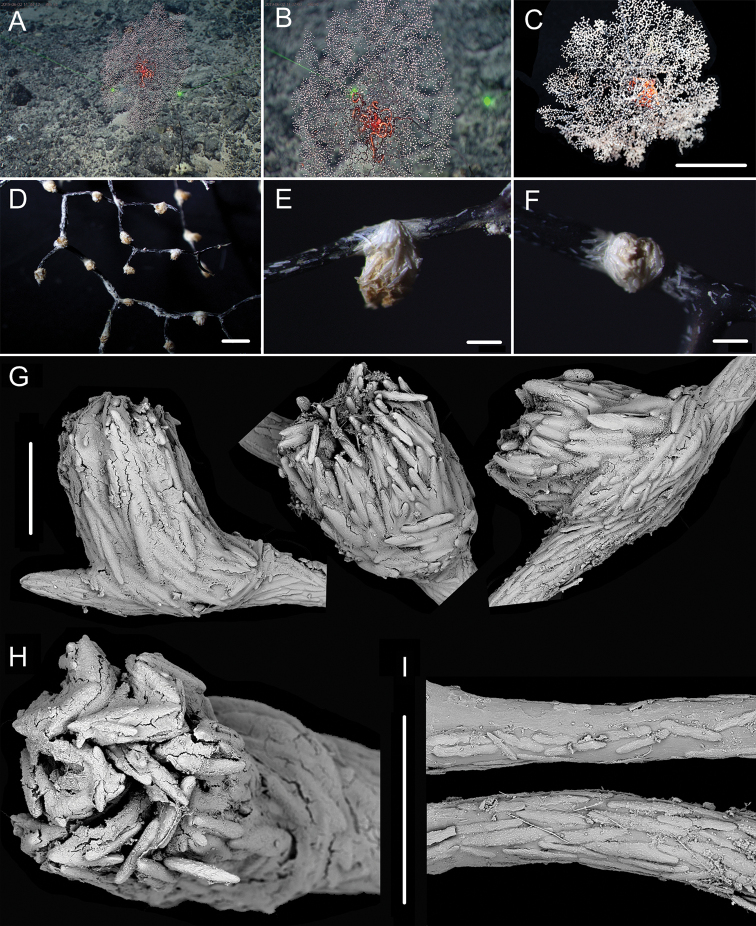
External morphology and polyps of *Metallogorgia macrospina* MBM286487 **A** the colony *in situ*. Laser dots spaced at 33 cm used for scale **B** close-up of branches *in situ***C** the colony after collection **D** a branch under a light microscope **E, F** a single polyp under a light microscope **G** three polyps under SEM**H** head of a polyp under SEM**I** coenenchyme under SEM. Scale bars: 10 cm (**C**), 2 mm (**D**), and 500 μm (**E–I; H, I** at the same scale).

**Figure 14.**
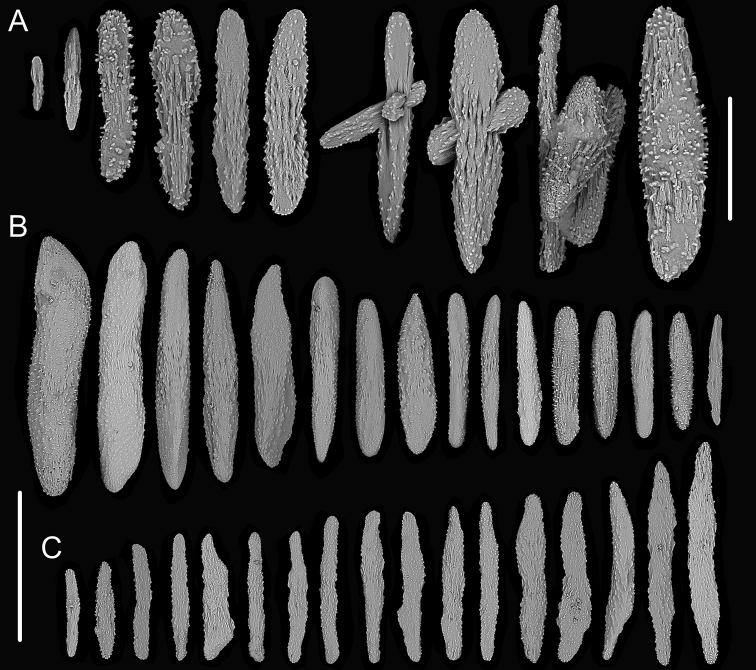
Sclerites of *Metallogorgia macrospina* MBM286487 **A** some small rods in polyps **B** larger rods in polyps **C** rods and elongated scales in coenenchyme. Scale bars: 100 μm (**A**), and 300 μm (**B, C** at the same scale).

#### Genus Pseudochrysogorgia

Taxon classificationAnimaliaAlcyonaceaChrysogorgiidae

Pante & France, 2010

E89F39BC-9FC9-57F4-972A-098702CB1805

##### Diagnosis

**(based on Pante and France 2010).** Main stem monopodial or slightly zigzagging with branches subdivided dichotomously in multiple planes, forming a bottlebrush-shaped colony. Most polyps leaning distad and neck narrower than head. Sclerites slightly ornamented, in the form of plates, scales and rods. When polyp not leaning distad, sclerites arranged obliquely on the polyp body. When polyps leaning distad, sclerites mostly arranged longitudinally (parallel to branch) on the polyp body, placed obliquely in the area of neck, and arranged longitudinally on head and along the back of tentacles. Scales and plates in branch coenenchyme mostly parallel to main branch axis.

##### Type species.

*Pseudochrysogorgia bellona* Pante & France, 2010.

##### Distribution.

Southwest Pacific (Coral Sea and northeast of New Zealand), 800–1462 m depth (Pante and France 2010, Pante et al. 2012).

**Figure 15.**
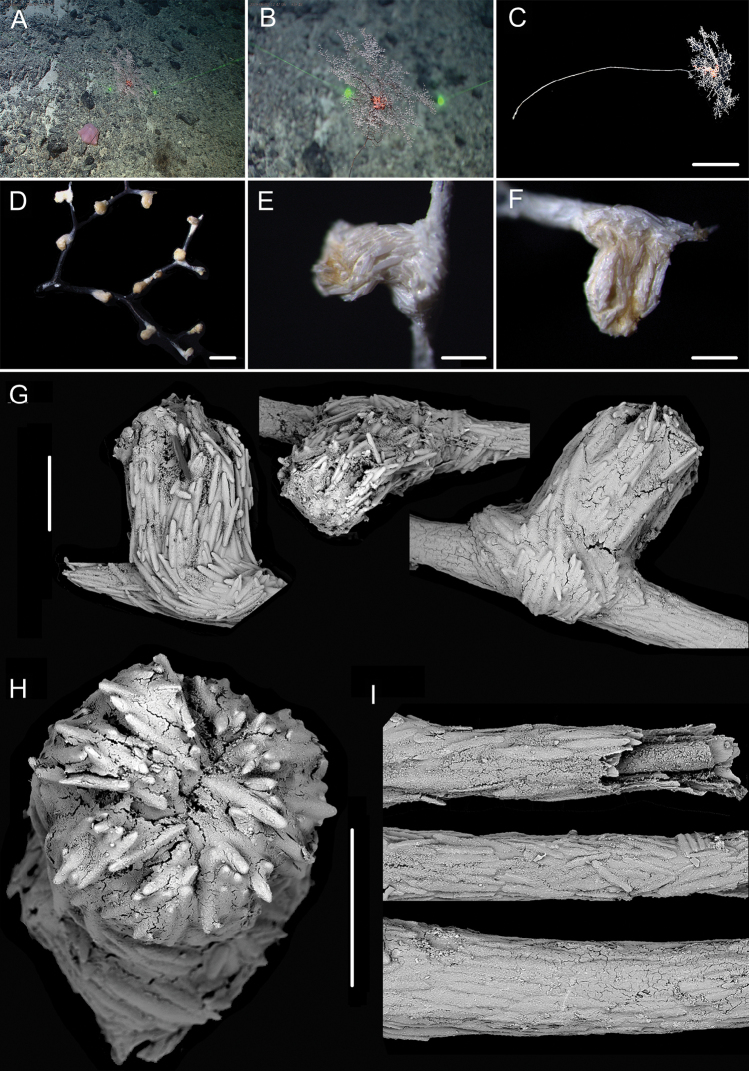
External morphology and polyps of *Metallogorgia macrospina* MBM286488 **A** the colony *in situ*. Laser dots spaced at 33 cm used for scale **B** close-up of branches *in situ***C** the colony after collection **D** a branch under a light microscope **E, F** a single polyp under a light microscope **G** three polyps under SEM**H** head of one polyp under SEM**I** coenenchyme under SEM. Scale bars: 10 cm (**C**); 2 mm (**D**), and 500 μm (**E–I; H, I** at the same scale).

**Figure 16.**
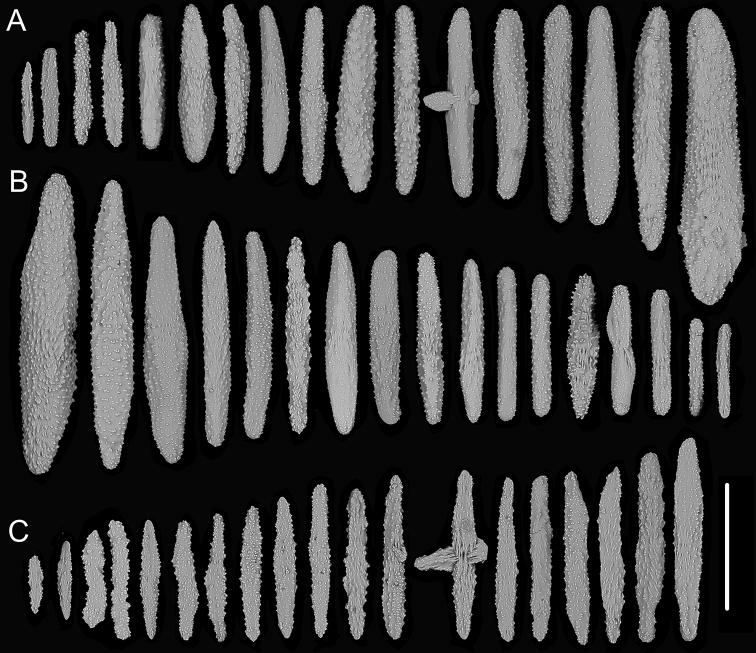
Sclerites of *Metallogorgia macrospina* MBM286488 **A** rods in tentacles **B** rods in the polyp-body wall **C** rods and elongated scales in coenenchyme. Scale bar: 200 μm (all images at the same scale).

**Figure 17.**
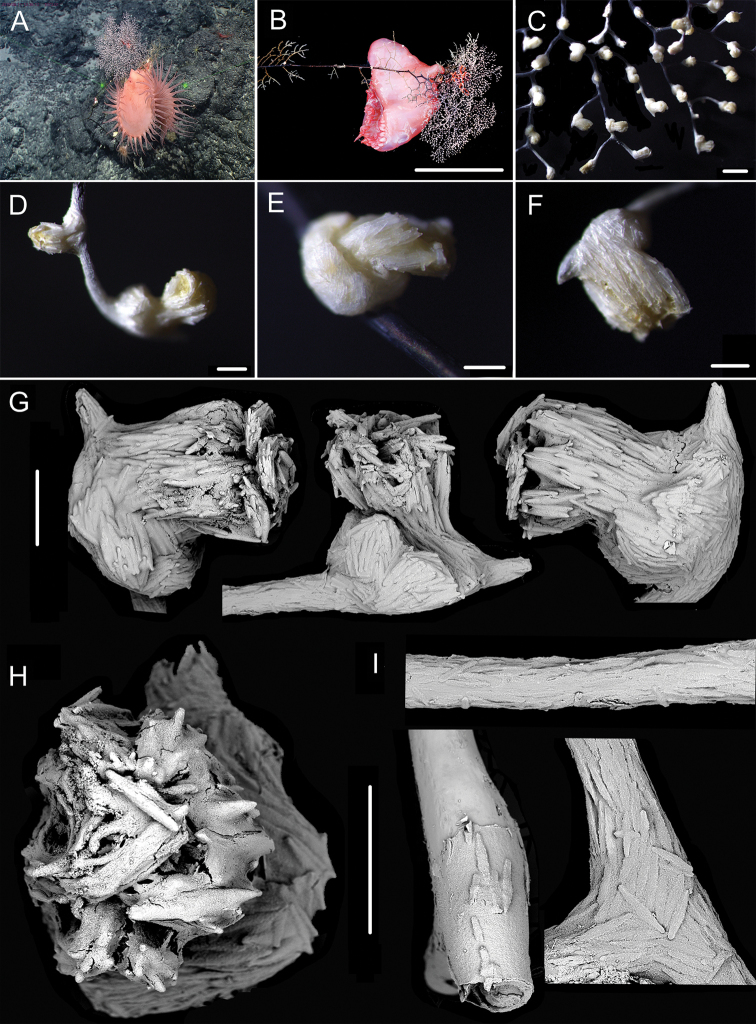
External morphology and polyps of *Metallogorgia macrospina* MBM286489 **A** the colony *in situ*. Laser dots spaced at 33 cm used for scale **B** the colony after collection **C** a branch under a light microscope **D** a terminal branchlet under a light microscope **E, F** a single polyp under a light microscope **G** three polyps under SEM**H** head of one polyp under SEM**I** coenenchyme under SEM. Scale bars: 10 cm (**B**), 2 mm (**C**), and 500 μm (**D–I; H, I** at the same scale).

**Figure 18.**
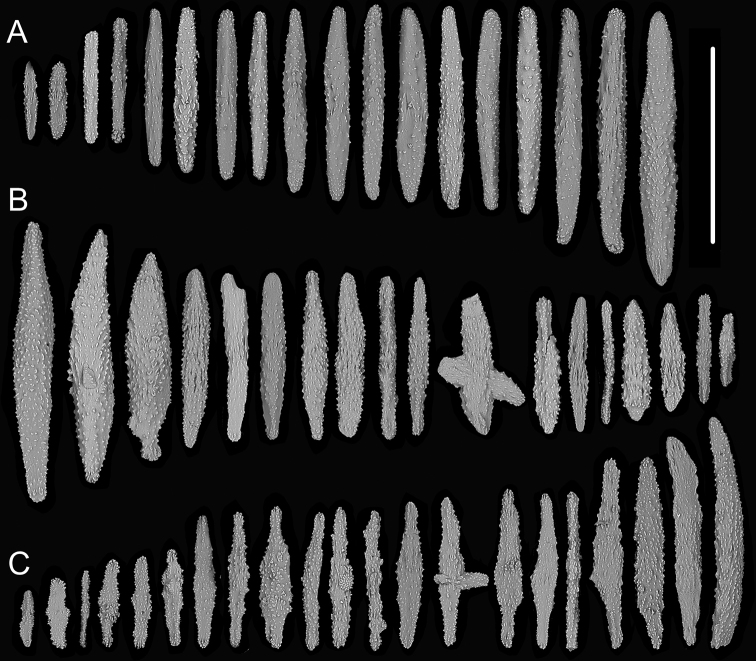
Sclerites of *Metallogorgia macrospina* MBM286489 **A** rods in tentacles **B** rods in the polyp-body wall **C** rods and elongated scales in coenenchyme. Scale bar: 300 μm (all at the same scale).

#### Pseudochrysogorgia bellona

Taxon classificationAnimaliaAlcyonaceaChrysogorgiidae

Pante & France, 2010

3D4DB35B-1924-53F2-A308-141F60998E1D

**Figure 19.**
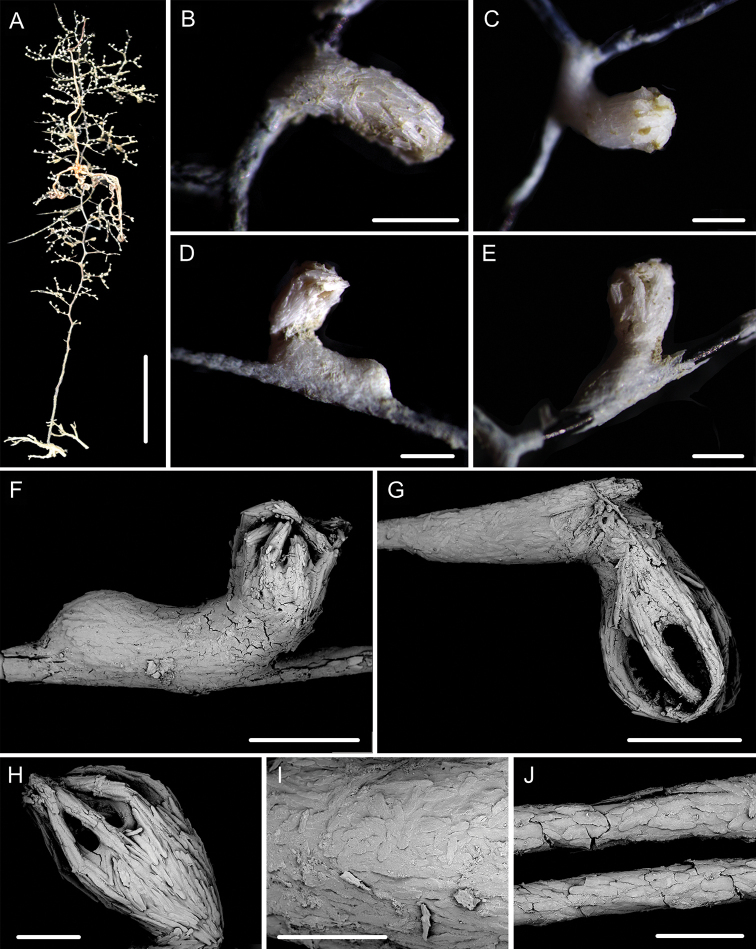
External morphology and polyps of *Pseudochrysogorgia bellona***A** the colony after collection **B–E** a single polyp under a light microscope **F, G** a single polyp under SEM**H** head of one polyp under SEM**I** basal polyp body under SEM**J** coenenchyme under SEM. Scale bars: 10 cm (**A**), 1 mm (**B–G**), and 500 μm (**H–J**).

**Figure 20.**
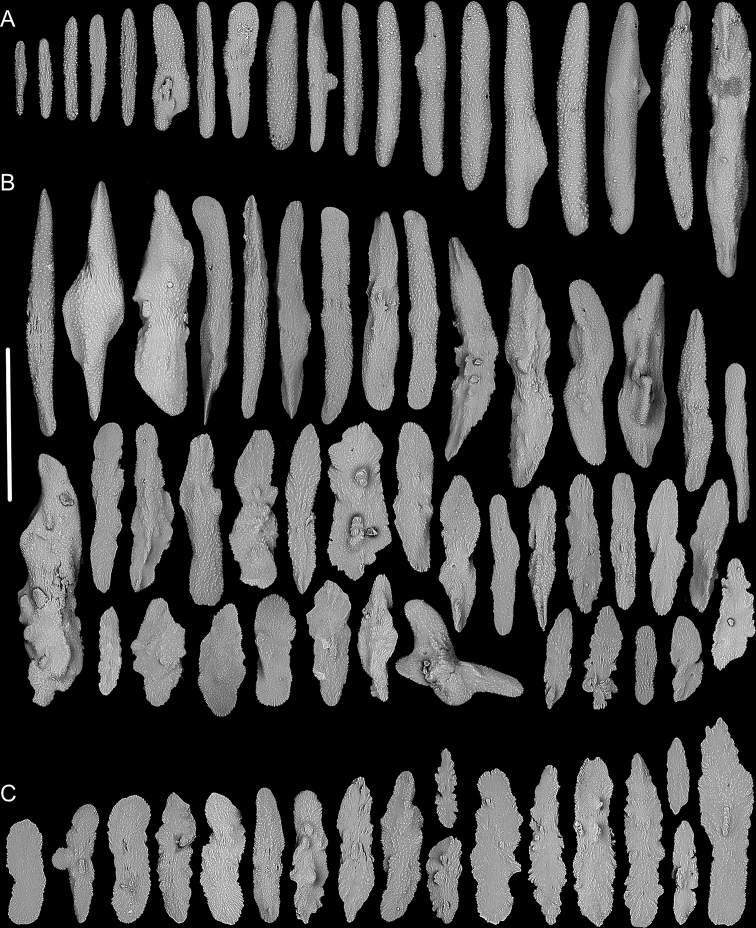
Sclerites of *Pseudochrysogorgia bellona***A** rods in tentacles **B** rods and scales in the polyp-body wall **C** scales in coenenchyme. Scale bar: 300 μm (all images at the same scale).

Figures 19 , 20 ; Table 3

Pseudochrysogorgia bellona Pante & France, 2010: 595–612.

##### Type locality.

Bellona Plateau, New Caledonia, 800–923 m depth (Pante and France 2010).

##### Voucher specimen.

MBM286490, the Ganquan Plateau in the South China Sea, 586–910 m.

##### Description.

No whole colony was obtained, thus the description was based on a picture (Figure 19A) and a few frozen fragments. Colony ca. 50 cm long and 11 cm wide with dendritic holdfast ca. 12 cm long. Main stem more zigzagging. Branching sequences unknown. Branches subdivided dichotomously, up to sixth order with distance between adjacent branches 10–25 mm. Polyps cylindrical, or with an expanded body base and obviously narrow neck, 1.0–2.5 mm long and 0.5–2.5 mm wide at bases, with tentacles up to 1 mm long (Figure 19B–G).

Rods with many small warts on surface, usually with two round ends and relatively less ornamentation, longitudinally arranged along the back of tentacles, and measuring 142–598 × 11–84 µm (Figures 19H, 20A). Sclerites in the polyp-body wall including rods, usually with one sharp end, and scales, slightly ornamented, some of them thick and elongated, and measuring 150–620 × 31–127 μm. When the polyp not leaning distad, rods and elongated scales arranged obliquely in the polyp-body wall. While polyps leaning distad, scales mostly arranged longitudinally (parallel to the branch) in upper of body, rods and scales placed obliquely in neck region, and arranged longitudinally on head and the base of tentacles (Figures 19I, 20B). Scales slightly ornamented, mostly parallel to main branch axis in coenenchyme, and measuring 115–397 × 16–150 μm (Figures 19J, 20C).

##### Distribution.

Bellona Plateau, Coral Sea and Otara Seamount, at southern tip of the Kermadec Ridge (Pante and France 2010, Pante et al. 2012); and South China Sea (present study), 586–1462 m depth.

##### Remarks.

*Pseudochrysogorgia bellona* Pante & France, 2010 resembles the species of *Chrysogorgia* Duchassaing & Michelotti, 1864 by having dichotomously subdivided branches arising from the main stem in a spiraling fashion and forming a bottlebrush-shaped colony. However, it differs in the monopodial or more zigzagging stem (vs. almost sympodial, except *Chrysogorgia abludo* Pante & Watling, 2012 and *C. dendritica* Xu, Zhan & Xu, 2020) and obviously different polyps (Xu et al. 2020). Furthermore, *P. bellona* is similar to species of *Metallogorgia* Versluys, 1902 in its monopodial colony and has a close genetic distance as well. However, *P. bellona* can be separated by its polyps usually with obviously narrow neck (vs. none), dendritic holdfast (vs. discoid) and sclerites including plates, scales and rods with more ornamentation (vs. almost rods and scales with little ornamentation) (Pante and France 2010). In our specimen, the rods in tentacles are more regular usually with two round ends and less ornamentation. The elongated thick scales are more abundant in coenenchyme, and plates are scarcer than the type colony. We suggest that these differences are population-dependent.

###### Molecular sequences, genetic distances, and phylogenetic analyses

**Figure 21.**
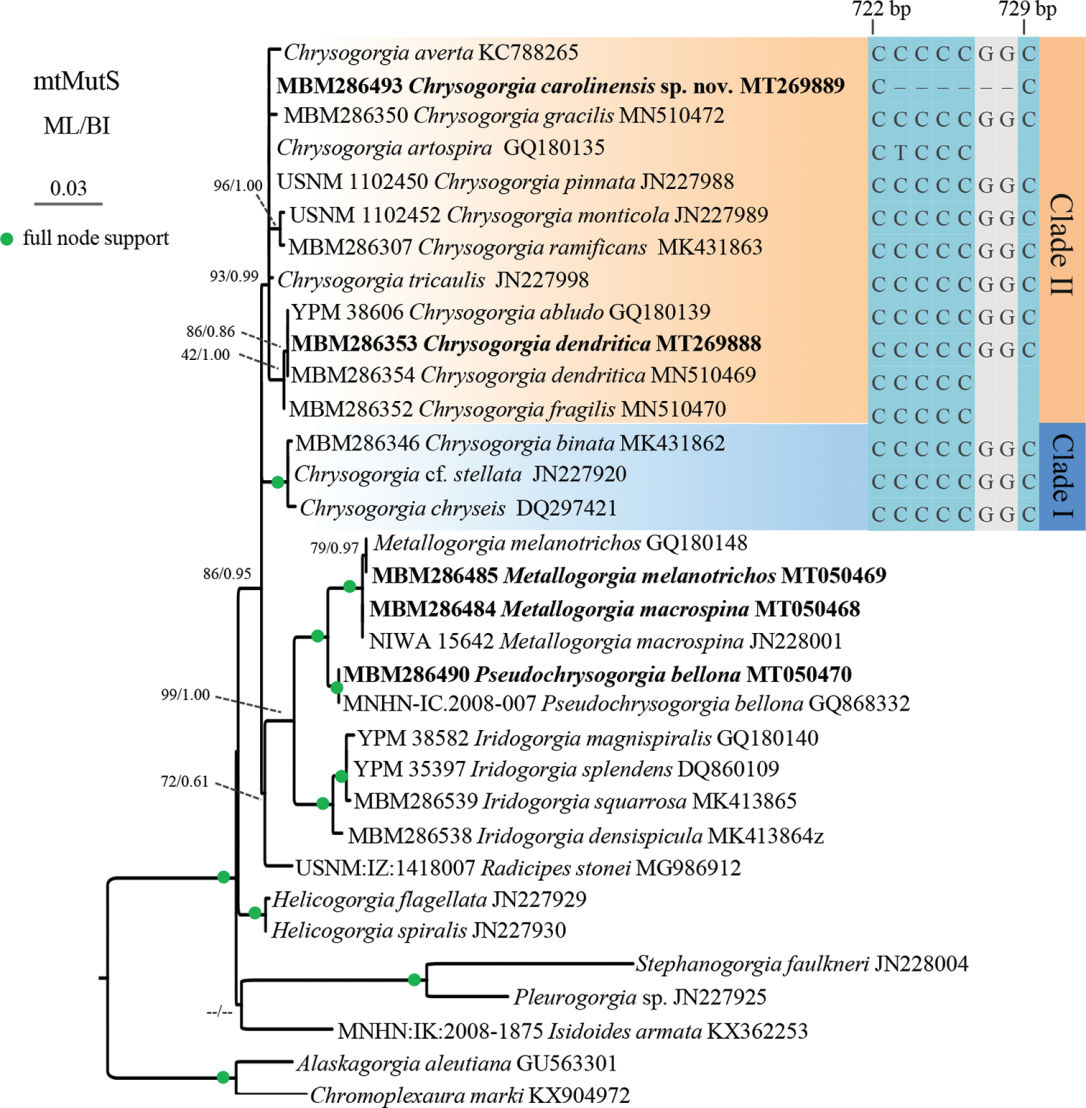
Maximum likelihood (ML) tree inferred from the mtMutS sequences of *Chrysogorgia*, *Metallogorgia*, *Pseudochrysogorgia* and related species. Numbers at the nodes represent the ML bootstrap values and the posterior probability values of Bayesian analysis (BI). Newly sequenced species are in bold. Support ≤ 50%/0.50 is shown as ‘--’. Alignment flatfile showed the mtMutS deletion for *Chrysogorgia carolinensis* sp. nov. with “–”, using *C. averata*KC788265 as a reference.

The conspecific sequences for each newly sampled species were identical, and only the holotype sequence was deposited in GenBank and analysed here. The accession number and the length are as follows: MT269889, 686 bp for *Chrysogorgia carolinensis* sp. nov., MT269888, 693 bp for *C. dendritica*, MT050468, 691 bp for *M. macrospina*; MT050469, 695 bp for *M. melanotrichos*, MT050470, 690 bp for *P. bellona*. The alignment dataset comprised 629 nucleotide positions. Compared to the other *Chrysogorgia* species, *C. carolinensis* sp. nov. has six-nucleotide deletion in the mtMust sequence (Figure 21). There was no intraspecific variability, while the interspecific distances ranged from zero to 2.42% (Table 4). The genetic distances between the new species *C. carolinensis* sp. nov. and its congeners were in range of 0–2.42%, and those between *C. dendritica* and its congeners were in the range of 0–1.63%. To date, only three species are available for the genetic distance and phylogenetic analysis for *Metallogorgia* and *Pseudochrysogorgia*. The mtMutS genetic distances between *M. macrospina* and *M. melanotrichos* was 0.16%, while there was no intraspecific variation within the two species and *P. bellona* (Table 4). The genetic distances between *Metallogorgia* and *Pseudochrysogorgia* were 1.94% and 2.10%.

**Table 4.** The uncorrected pairwise distances at mtMutS between *Chrysogorgia*, *Metallogorgia* and *Pseudochrysogorgia* species/populations.

	Species/populations	1	2	3	4	5	6	7	8	9	10	11	12	13	14	15	16	17
1	*C. abludo*GQ180139, JN227999	–																
2	*C. dendritica*MN510469, MT269888	0	–															
3	*C. fragilis* MN510470	0.16%	0.16%	–														
4	*C. gracilis* MN510472	0.97%	0.97%	1.13%	–													
5	***C. carolinensis* sp. nov.** MT269889	0.81%	0.81%	0.65%	0.33%	–												
6	*C. artospira*GQ180132–GQ180135, GQ353317	0.81%	0.81%	0.65%	0.48%	0	0											
7	*C. pinnata* JN227988	0.81%	0.81%	0.65%	0.48%	0	0	–										
8	*C. tricaulis*JN227998, JN227990, JN227991, GQ180123–GQ180131, EU268056	0.97%	0.97%	0.81%	0.65%	0.16%	0.16%	0.16%	0									
9	*C. averata*KC788265, GQ180136	1.13%	1.13%	0.97%	0.81%	0.33%	0.32%	0.32%	0.48%	0								
10	*C. monticola* JN227989	1.45%	1.45%	1.29%	1.13%	0.65%	0.65%	0.65%	0.81%	0.97%	–							
11	*C. ramificans* MK431863	1.45%	1.45%	1.29%	1.13%	0.65%	0.65%	0.65%	0.81%	0.97%	0.32%							
12	*C. chryseis*JN227992, DQ297421	2.42%	2.42%	2.26%	2.10%	1.63%	1.62%	1.62%	1.78%	1.94%	2.26%	2.26%	–					
13	*C. binata* MK431862	2.42%	2.42%	2.26%	2.10%	1.63%	1.62%	1.62%	1.78%	1.94%	2.26%	2.26%	0.48%	–				
14	C. cf. stellata JN227920	2.26%	2.26%	2.10%	1.94%	1.46%	1.45%	1.45%	1.62%	1.78%	2.10%	2.10%	0.32%	0.16%	–			
15	*Metallogorgia macrospina*MT050468, JN228001, JN227906	5.33%	5.33%	5.17%	4.85%	4.55%	4.52%	4.52%	4.68%	4.52%	5.17%	5.17%	5.17%	5.17%	5.01%	0		
16	*M. melanotrichos*MT050469, GQ868333, GQ868339, GQ868340, GQ180146–GQ180155, GQ180156–GQ180158, GQ180162, GQ180163, GQ353314, EU268057, DQ297423	5.49%	5.49%	5.33%	5.01%	4.72%	4.68%	4.68%	4.85%	4.68%	5.33%	5.33%	5.33%	5.33%	5.17%	0.16%	0	
17	*Pseudochrysogorgia bellona*MT050470, GQ868331, GQ868332	4.04%	4.04%	3.88%	3.55%.	3.25%	3.23%	3.23%	3.39%	3.55%	3.88%	3.88%	4.52%	4.52%	4.36%	1.94%	2.10%	0

The ML and BI phylogenetic trees are identical in topology, and thus only the former with the both support values is shown (Figure 21). In both trees, *Metallogorgia*, *Pseudochrysogorgia*, *Iridogorgia*, *Radicipes* and *Chrysogorgia* formed a “monophyletic Chrysogorgiidae clade’’ (MCC) sensu Pante et al. (2012) with moderate to high support (ML 86%; BI 0.95). The *Chrysogorgia* species were separated into two main clades (Clade I and II) with high support values, which is consistent with Xu et al. (2019, 2020). Clade I includes the sister species *C. binata*, C. cf. stellata and *C. chryseis*, and Clade II contains all the rest species. The species *C. dendritica* and *C. abludo* formed a sister subclade, followed by *C. fragilis*. *Chrysogorgia carolinensis* sp. nov. formed a sister subclade with the cluster *C. ramificans* + *C. monticola*, the group *C. dendritica* + *C. abludo* + *C. fragilis* and the rest species within Clade II. *Metallogorgia melanotrichos* and *M. macrospina* formed sister clade, followed by *P. bellona* with full node support (Figure 21).

## Discussion

Both the morphology and molecular phylogenetic analysis support the assignment of the new species to the genus *Chrysogorgia* Duchassaing & Michelotti, 1864. The barcoding analysis of mtMutS is considered as the first step in molecular identification of octocorals (McFadden et al. 2011; Pante et al. 2012). However, the mtMutS genetic distances within *Chrysogorgia* are relatively low, and there is no barcoding gap (intraspecific zero vs. interspecific 0–2.42%) for species identification, as shown by Xu et al. (2020). Thus, the discrimination of the closely related species was mainly relied on other molecular evidence and morphological characters. Alternatively, single mutations on mtMutS can be used to separate *Chrysogorgia* species (Pante and Watling 2012; Pante et al. 2015; Xu et al. 2020). The mtMutS sequence of *C. carolinensis* sp. nov. has six deletion mutations compared to those of its congeners (Figure 20), supporting the establishment of the new species. Furthermore, the closely related species *C. carolinensis* sp. nov., *C. artospira* and *C. pinnata* are easily separated by the 1/3L branching sequence (vs. 2/5L and flabellate, respectively) and amoeba-shaped scales at the basal polyp body (vs. regular scales and rods, respectively) (Cairns 2007; Pante and Watling 2012). No genetic variability was observed between *C. dendritica* and the closely related species *C. abludo*. However, the former is morphologically distinctly different from the latter by the presence of irregular sclerites in the polyp-body wall (often branched and amoeba-shaped with more lobed edges vs. relatively regular with less lobed edges; Pante and Watling 2012; Xu et al. 2020).

Colonial branching pattern was regarded as one of the diagnostic characters to distinguish chrysogorgiid octocorals (Pante et al. 2012). However, it should be carefully applied to the identification of *Metallogorgia* species, since polymorphism of the colony shape is known for *M. melanotrichos* during its life cycle (Mosher and Watling 2009). In the present study, one juvenile and three adults of *M. macrospina* Kükenthal, 1919 were collected and identified. There are also obvious morphological differences between the juvenile and the adults in the colony shape and branch pattern (see before, Figures 11C, 15C). Nevertheless, like the case of *M. melanotrichos*, their conspecific assignment can be determined by the same polyps and sclerites, and the MutS gene sequences (Figures 11–18, 21). On the other hand, the *M. melanotrichos* and *M. macrospina* juveniles rather than the adults, are most similar to the *Pseudochrysogorgia* in having lateral branches subdivided dichotomously and not forming a sympodium, and a slightly bottlebrush-shaped colony, mirroring their sister relationship showed by the phylogenetic trees (Figures 11C, 21). Seemingly, the colony branch morphogenesis may be useful to infer the phylogenetic relationship for chrysogorgiids, and the colonial branching pattern may be an apomorphy for chrysogorgiids.

To include the juvenile morphology, we slightly extend the diagnosis of the genus *Metallogorgia* on the basis of Versluys (1902) and Kükenthal (1919): Colonies with distinctly monopodial stem giving rise to a few lateral branches. Branches arising distally in adults, while arising on the lateral of stem randomly in juveniles. Branch subdivided dichotomously, with branchlets either forming a sympodium in one plane in adults, or in multiple planes in juvenile. Branching part monopodial forming an approximate planar layer or a slight bottlebrush shape, or sympodial forming a spiral or tree shape. Axis round, hard and strong with a smooth surface and an extremely pronounced metallic luster. Polyps cylindrical without obvious neck, relatively little to the size of branches they sit on and well separated from one another, covered with abundant and dense sclerites. Coenenchyme thin, most with a few sclerites or not differentiated well into this layer. Sclerites in the main form of rods, spindles and scales, with little ornamentation.

Furthermore, based on the present morphological descriptions, we provide a preliminary key to full grow grown specimens of the genus *Metallogorgia* Versluys, 1902.

### 

**Table:** 

1	Only scales present in coenenchyme	**2**
–	Rods present in coenenchyme	**3**
2	Rods and spindles present in polyp body wall	***M. tenuis***
–	Rods and scales present in polyp body wall	***M. melanotrichos***
3	Plates present in tentacles and only rods in coenenchyme	***M. splendens***
–	Plates absent in tentacles, rods and scales present in coenenchyme	***M. macrospina***

## Supplementary Material

XML Treatment for Chrysogorgia

XML Treatment for Chrysogorgia dendritica

XML Treatment for Chrysogorgia carolinensis

XML Treatment for Metallogorgia

XML Treatment for Metallogorgia melanotrichos

XML Treatment for Metallogorgia macrospina

XML Treatment for Pseudochrysogorgia

XML Treatment for Pseudochrysogorgia bellona
